# Stress, health, noise exposures, and injuries among electronic waste recycling workers in Ghana

**DOI:** 10.1186/s12995-018-0222-9

**Published:** 2019-01-10

**Authors:** Katrina N. Burns, Stephanie K. Sayler, Richard L. Neitzel

**Affiliations:** Department of Environmental Health Sciences, University of Michigan, 1415 Washington Heights 6611 SPH I, Ann Arbor, MI 48109 USA

**Keywords:** Electronic waste recycling, Occupational injuries, Health, Noise exposure, Stress

## Abstract

**Background:**

Electronic waste (e-waste) recycling workers in low and middle-income countries have the potential for occupational injuries due to the nature of their work at informal e-waste sites. However, limited research exists on stress, noise, occupational injuries, and health risks associated with this work environment. This study evaluated injury experience, noise exposures, and stress risk factors among e-waste workers at the large recycling site in the Agbogbloshie market, Accra, Ghana.

**Methods:**

Participants completed a survey addressing their work, health status, stress, exposures to several occupational hazards (including noise), use of personal protective equipment at work, and injury experience. A subset of participants also completed personal noise dosimetry measurements. Poisson regression was used to evaluate the association between the number of injuries experienced by participants and various factors evaluated in the survey.

**Results:**

Forty-six male e-waste workers completed the survey, and 26 completed a noise dosimetry measurement. Participants experienced an average of 9.9 ± 9.6 injuries per person in the previous 6 months (range: 1–40). The majority of injuries were lacerations (65.2%), and the most common injury location was the hand (45.7%). Use of personal protective equipment was rare. The mean time-weighted average noise level was 78.8 ± 5.9 dBA. Higher perceived stress, greater age, poorer health status, not using gloves, and involvement in dismantling activities were associated with an increased number of injuries. After controlling for each of these risk factors, perceived stress level and perceived noise exposure were associated with a significantly greater number of injuries.

**Conclusions:**

Our study identified a large number of injuries among informal e-waste recyclers, and we found that higher levels of perceived stress and perceived noise were associated with an increased number of occupational injuries, even after controlling for other injury risk factors.

## Background

Electronic waste (e-waste) consists of discarded cell phones, computers, appliances, and other electrical or electronic products, and electronic waste recycling involves the salvaging of these items for repair or for extraction of valuable metals and components. As of 2016, a total of 44.7 million tons of e-waste had been created globally, and that number is expected to grow to 52.2 million by 2021 [10]. Due to the high consumer demand for the latest generation of electronics, and subsequent discarding of older electronics, the amount of e-waste being created is increasing substantially over time [24, 30, 45]. The high e-waste recycling and disposal costs in high-income countries has led to the exportation of e-waste to low- and middle-income countries (LMICs) where labor costs are lower, but where resources to recycle and dispose of the products in a safe and sustainable manner are limited or absent [44]. The 1989 Basel Convention was developed to prevent the inter-country movement of hazardous waste, but some high-income countries, such as the United States, have not ratified the convention and continue to export used, obsolete, and often unrepairable electronic equipment as “donations” to countries throughout Africa and Asia, where they are recycled by informal workers [50]. This informal recycling scheme creates important economic opportunities for impoverished workers and communities in LMICs and recovers valuable raw materials from e-waste, preventing these materials from being discarded, but the processes used may result in unnecessarily high occupational risks [3, 9]. Unfortunately, informal e-waste recycling sites are not well-regulated by local governments and many may have no occupational health and safety oversight.

Informal e-waste recycling workers collect, sort, and repair or dismantle e-waste using crude de-manufacturing methods and very basic, non-specialized hand tools. Workers often remove plastic or rubber coatings from wires and other valuable metal components for resale (the final goal of e-waste recycling activities) by burning the e-waste materials. All of these tasks are often performed without the benefit of safe working procedures, personal protective equipment (PPE), sanitation facilities, or safety training, all of which are common features in formal work settings in high-income nations [18, 48]. This can place e-waste workers at an increased risk of injury, exposure to noise and subsequent noise-induced hearing loss, and multiple other adverse health effects associated with their work [5, 15, 39, 54]. In addition to the adverse physical health impacts, workers may also suffer from personal and occupational stress due to factors often affecting vulnerable (and frequently migrant) e-waste workers [15]. Regulatory attention and efforts to improve occupational health among e-waste recycling workers could result in safer working and living conditions, a living wage [8, 11, 35, 43], and better access to PPE, but additional research is needed to better characterize occupational health needs among these workers.

The Agbogbloshie market in Accra, Ghana, offers an excellent example of informal e-waste recycling. After over 10 years of accepting and recycling electronic waste from Europe, North America, and Asia [6], Agbogbloshie has become one of the most polluted informal e-waste recycling sites in the world [23]. The informal e-waste site at Agbogbloshie consists of primarily workers who have traveled to the site from northern Ghana and, to a lesser extent, other West African countries in search of work. At the time of our study this site was effectively unregulated; there was no formal oversight for the e-waste recycling workers at the site, and all workers were essentially independent contractors. The payment structure used at the site was based on the amount by weight of valuable metals (primarily copper) that each worker was able to recover from the e-waste. To the best of our knowledge, the Greater Accra Scrap Dealers Association, which provided overall leadership to work operations at the site, but which did not directly employ any e-waste recycling workers, did not advocate for the workers in any formalized capacity or control employment conditions at the site.

In order to address the multiple occupational exposures among this vulnerable population of recycling workers, we examined the relationship between e-waste work activities, stress, noise exposures, and injury experience. While a number of studies have focused on the relationship between noise and injuries [16, 17, 19, 33, 36, 37], there is a paucity of studies on injuries among e-waste recycling workers [18], and those that have been conducted have not examined work activities in detail [27]. Our study had two hypotheses: first, that higher perceived stress levels would be associated with higher injury risk, and second, that higher noise exposures (evaluated both objectively and subjectively) would be associated with higher injury risk. Our study evaluated exposures and injury outcomes without defining or exploring an a priori-defined injury mechanism.

## Methods

### Overview

Our previously described data collection methods [15] will be briefly summarized here. All research procedures were approved by the University of Michigan Institutional Review Board (HUM00084062) and the University of Ghana Institutional Review Board at the Noguchi Memorial Institute for Medical Research (NMIMR-IRB CPN 070/13-14). A research team composed of students, staff, and faculty members from the University of Michigan and the University of Ghana-Legon collected the data in May 2014.

### Recruitment

E-waste recyclers 18 years of age and older who worked at Agbogbloshie during the study were recruited to participate in the study. We conducted recruitment with the approval and assistance of the Chairman of the Greater Accra Scrap Dealers’ Association. Recruiting was conducted with the assistance of hired translators, and scheduled for approximately 1 h in the mornings and 2–3 h in the afternoons of days in which we collected data. Our goal was to recruit a convenience sample of 60 e-waste recycling workers, recruited through word of mouth and by our translators actively approaching individuals onsite to assess their interest. Workers participated in this study over a single day each, but were not required to be actively working on their day of participation. Each participant was approached in person and read a recruitment script and the informed consent in their native language. Interested individuals signed or provided an ink thumbprint on the informed consent form for official enrollment in the study. Participants received 9 GHS (about 3 USD) and a 3 GHS (about 1 USD) snack as a thank-you for their participation.

### Survey

All participants completed a comprehensive interview administered in the language of their preference by our hired interpreters. The interview consisted of questions on demographics (e.g., age, religion, marital status, education, time living in Agbogbloshie, and income); health-related behaviors and outcomes (e.g., smoking status, self-reported health status); and personal stress factors, as measured by a subscale of Cohen’s Perceived Stress Scale (PSS, [21]) with a total of 16 points possible and higher scores indicating greater stress. The PSS survey has been translated and validated in a number of countries, but not, to our knowledge, in Ghana. Occupational information was also collected through the survey, including work activities, work duration, use of personal protective equipment (PPE), information on job demands and working conditions, and the number of injuries received during recent e-waste recycling activities. Work activities were defined after making initial observations of ongoing work at the site and with the assistance of the Chairman of the Greater Accra Scrap Dealers’ Association. The final categories developed were: burning of e-waste, collecting e-waste from consumers or businesses, collecting e-waste after burning, dismantling e-waste, lead acid battery recycling, lead smelting, removing wire coverings, repairing e-waste, and sorting e-waste. Occupational stress scores (OSS) were calculated on 28-point scale based on occupational stress-related survey questions; as with the perceived stress scale, higher values indicated higher levels of occupational stress. The frequency of exposure to noise, intended to represent participants’ “typical” exposure, was subjectively assessed on a five-point scale, with categories of “Never,” “Almost never,” “Sometimes,” “Fairly often,” “Very often,” or “Don’t Know.”

### Noise measurements

While the perceived frequency of noise exposure question on the survey provided information about typical exposures, we also objectively measured personal noise exposures using ER-200D personal noise dosimeters (Etymotic Research Inc. Elk Grove Village, IL, USA). The dosimetry data were intended to compliment the subjective data by providing quantified estimates of noise exposure levels overall, as well as during particular work and non-work activities. These data were also collected to allow for assessment of the potential relationship between objective noise levels and injury risk. The devices were configured to measure the equivalent continuous average noise level (L_EQ_) according to the exposure limit for community noise recommended by the World Health Organization: A-weighting, slow time response, 3 dB time-intensity exchange rate, 70 dB threshold, and 75 dB criterion level [13]. The measurement range of the noise dosimeter was 70–130 dBA. Average and maximum noise exposures were data logged every 3 min 45 s (the default and only data-logging interval length available for these dosimeters) for up to 24 h. Exposures during work activities were compared to the 85 dBA exposure limit used for occupational noise exposures in most countries around the world [49].

### Data cleaning and statistical analysis

Study data were cleaned using methods we have described previously [15]. All statistical analyses were performed using SPSS Version 24.0 (IBM SPSS Statistics for Windows, IBM Corp., Armonk, NY, USA). For workers who completed personal noise dosimetry, we computed 8-h time-weighted average (TWA, in dBA) exposures using Eq. 1, where *L*_*AEQ*_ is a 3.75-min average equivalent continuous noise level, *N* is the total number of 3.75-min intervals *i* in the measured shift, and 128 is the number of 3.75-min intervals in a 480-min shift.

1 $$ TWA=10\times {\mathit{\log}}_{10}\left[1/128{\int}_{i=1}^N1\times {10}^{L_{AEQ}/10}\right] $$

Descriptive univariate and bivariate analyses were conducted; for all inferential analyses, results were considered statistically significant where *p* < 0.05. We used multivariable Poisson regression [34] to model the relationship between the number of self-reported e-waste-related injuries and other occupational and non-occupational factors. Eq. 2 depicts the Poisson regression model, where the expected rate *(E)* of the number of injuries *(Y)* given the continuous and categorical predictor variables, *x*, equals the value of the effect on the predictors, *α*, added to the coefficient *β*^′^, or the multiplicative effect on the mean of *(Y)* as the result of *x*.

2 $$ \mathit{\log}\left(E\left(Y|x\right)\right)=\alpha +{\beta}^{\prime }x $$

We ran unadjusted (i.e., single predictor variable) and adjusted Poisson regression models on a number of potential injury risk factors. Variables tested in unadjusted models were chosen based on the participant responses to questions about work activities where they received the most injuries and the body sites reported where workers sustained injuries. Noise-related variables were selected due to the knowledge of the worksite conditions, worker responses to questions about noise exposure and a priori knowledge of noise related occupational injuries [4, 16, 17, 33, 38]. Each variable was tested individually in an unadjusted Poisson model to determine its effect on the outcome and on improvements in model fit (as assessed via the Akaike Information Criterion, AIC). A forward stepwise selection routine was used to select the final adjusted model. Two variables (age and self-reported health status) were forced into our adjusted model based on a priori assumptions derived from previous research [12, 22, 47], though it is important to note that these prior studies were conducted in formal (and not informal) work settings. However, recent studies on informal waste sites in Nigeria demonstrate a significant association of age to adverse outcomes of exposure and injury [41, 42].

We also performed a sensitivity analysis related to noise exposures. We repeated the final multivariable Poisson regression developed from all workers on the subset of workers who completed personal noise dosimetry, and replaced the self-reported noise exposure frequency variable with a variable representing measured noise exposure level in dBA.

## Results

### Demographics and health status

Demographic characteristics are shown in Table 1 for all participants (*N* = 46 e-waste workers) and the subset of workers who completed personal noise dosimetry (*N* = 26). We did not track the total number of potential participants approached to participate, and so cannot report a participation rate. Among workers who declined to participate, the most common reason given was an insufficient financial incentive. No significant differences were noted between the total and subset samples (data not shown). Participants had an average age of about 25 ± 6 years old, and had been a resident at the site for an average of 5 ± 3 years. Slightly over half of had no formal education, and a similar fraction described their general health as fair or poor. The majority of participants were migrants from Northern Ghana who had traveled to Accra to pursue employment opportunities (data not shown). Dagbani was by far the most commonly-used interview language (65% of participants), and a slight majority of interviews were conducted by interviewer 1.

**Table 1:** Demographics information (*n* = 46 e-waste recycling workers)

Continuous Variable	Units	*N*	Mean (SD)
Age	Years	46	25 (6.4)
Duration of residence	Years	46	5.7 (3.3)
Categorical variable	Category	*n*	%
Relationship Status	Divorced/separated	2	4
	Married	27	59
	Single	17	37
Education Level	None/Never	26	57
	Primary	9	20
	Middle/JSS	9	20
	Secondary/SSS	2	4
Self-reported health status	Excellent	4	9
	Very good	7	15
	Good	11	24
	Fair	20	44
	Poor	4	9
Interview language	Twi	1	2
	Dagbani	30	65
	English	2	4
	FraFra	1	2
	Dagbani and English	2	4
	Other	10	22
Interviewer	1	26	57
	2	20	43

### Work-related activities

Participants had worked at the site an average of 5.7 ± 3.3 years, and worked an average of approximately 10 h/day (Table 2). Workers reported participating in a wide variety of activities, and generally did not specialize in any activity. The most commonly-reported activities were dismantling e-waste (reported by 83% of participants) collecting e-waste (80%) and sorting e-waste (78%). The least-commonly reported activities included lead smelting (reported by 39% of participants) and removing wire coverings from e-waste (28%).

**Table 2:** Work Characteristics, Activities, and Exposures (*N* = 46 male e-waste workers)^a^

Continuous variable	Units	*N* workers	% workers	Mean	SD
Time in primary job	Years	43	94	5.7	3.3
Hours worked/day	Hours	42	91	10.5	2.3
Perceived Stress Score	Score (0 lowest to 16 highest)	46	100	9.9	2.9
Occupational Stress Score	Score (0 lowest to 28 highest)	43	94	18.9	5.1
Categorical variable	Category	*N* workers	% workers		
E-waste activities reported by workers (more than 1 activity could be reported by each worker)	Burning e-waste	34	74		
	Collecting e-waste from consumers and businesses	37	80		
	Collection e-waste after burning	35	76		
	Dismantling e-waste	38	83		
	Lead acid battery recycling	21	46		
	Lead smelting	18	39		
	Remove wire coverings from e-waste	34	28		
	Repairing e-waste	13	28		
	Sorting e-waste	36	78		
How much does noise annoy you?	Not at all	10	22	–	–
	A little or great deal	36	80	–	–
Perceived noise exposure	Less than very often	6	13	–	–
	Very often	40	87	–	–
Daily income (GHS)	<GHS 10	15	33	–	–
	GHS 11–20	17	37	–	–
	GHS > 21	14	30	–	–
Impairment that limits work		9	20	–	–
Received training prior to injury		20	44	–	–

^a^Not all categories add up to 26 or 46 due to missing data

The average score on the PSS and OSS was over 50% of the total possible points (PSS mean = 9.9 of 16 possible points, OSS mean = 19.7 of 32 possible points). Nineteen percent of participants reported impairments that limited their work abilities, and a minority of workers (43.5%) reported receiving any safety training prior to performing work that resulted in injury. Thirty-eight percent of the participants made less than 10 Ghanaian Cedis (about 2.20 USD) per day; for comparison, the average daily wage in Ghana during the time period of this study was 6 Ghanaian Cedis (about 1.30 USD, [1]). While seniority (i.e., more time on the job) is often associated with greater pay, the correlation between time at the site and pay was poor and insignificant.

### Self-reported and measured noise exposures

The vast majority of workers (87%) reported frequent exposure to high noise on the job, and also reported being frequently bothered by this noise (78.3%, Table 2). Among the 26 workers with personal dosimetry, the average TWA for noise was 78.8 ± 5.9 dBA. Approximately 15% of TWA exposures exceeded the recommended 85 dBA exposure limit; all of these overexposures were associated with dismantling activities. Additionally, 73% of the workers reporting frequent exposure to high noise did dismantling. The Spearman correlation coefficient between measured noise level (in dBA) and self-reported noise exposure frequency among the participants who completed personal noise dosimetry was not significant (*p* = 0.21).

### Injury experience and potential injury risk factors

**Fig. 1:**
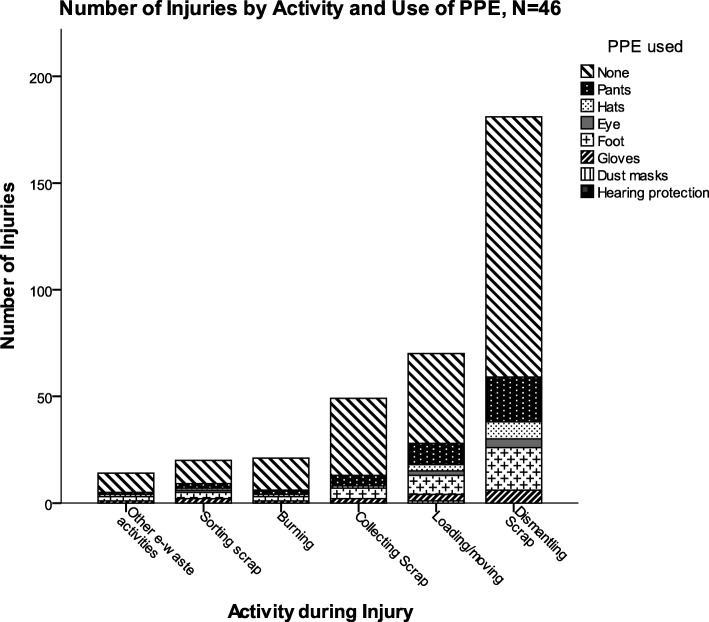
Number of injuries by work activity and use of personal protection equipment (*N* = 46 workers)

A total of 426 injuries in the prior 6 months were reported across all 46 participants. The average number of injuries was 9.9 ± 9.6 per participant, though the range of injuries was quite large (1–40 injuries per participant, Table 3). The total number of days of work missed due to occupational injuries was 193; the average number of missed workdays for injuries during this period was 4 ± 2.6 days, although few participants were hospitalized for their injuries (6.5%). Most of the reported injuries were lacerations (65.2%), and the most common injury locations was the hand (45.7%). The PPE categories most commonly used were pants and foot protection (defined here as shoes, as opposed to the open sandals commonly used by workers on the site). Few workers reported wearing gloves; only 26% reported glove use during dismantling or sorting of e-waste. Fisher’s exact tests of the associations between glove use and age, income, or seniority. No workers reported wearing hearing protection. The work activity associated with the most injuries was dismantling, an activity during which PPE use was rare (Fig. 1). PPE use was uncommon during all tasks, and at least 50% of injuries occurred when participants were not using any PPE.

**Table 3:** E-waste recycling injuries, activities, and use of personal protective equipment (*N* = 46 e-waste workers)

Variable	Category	Number	Percent	Mean	SD
Number of E-waste injuries in previous 6 months		43	94	9.9	9.6
Number of missed work days		18	39	10.7	9.0
Hospitalized for worst injury		3	7	–	–
Causes of E-waste related injuries	Burns	1	2	–	–
	Caught in-between	2	4	–	–
	Contact with sharp objects	20	44	–	–
	Struck and hit	13	28	–	–
	Other	3	7	–	–
Injury type	Cuts/lacerations/abrasions	30	65	–	–
	Burns/scalds	2	4	–	–
	Internal injury	3	7	–	–
	Other	2	4	–	–
Body Part Injured^a^	Arm	2	4	–	–
	Eyes	2	4	–	–
	Foot (toes)	7	15	–	–
	Hand(fingers)	21	46	–	–
	Head	2	4	–	–
	Leg (ankle, knee)	10	22	–	–
Activity at the time of Injury	Burning	1	2	–	–
	Collecting	6	12	–	–
	Dismantling	24	52	–	–
	Loading/moving	10	22	–	–
	Sorting	1	2	–	–
	Multiple activities	2	4	–	–
PPE generally worn for job	Dust Mask	1	2	–	–
	Eye protection	7	15	–	–
	Foot Protection	36	78	–	–
	Gloves	12	26	–	–
	Hats	11	25	–	–
	Pants	39	85	–	–

^a^Does not add up to total number of injuries due to multiple sites harmed per event

**Fig. 2:**
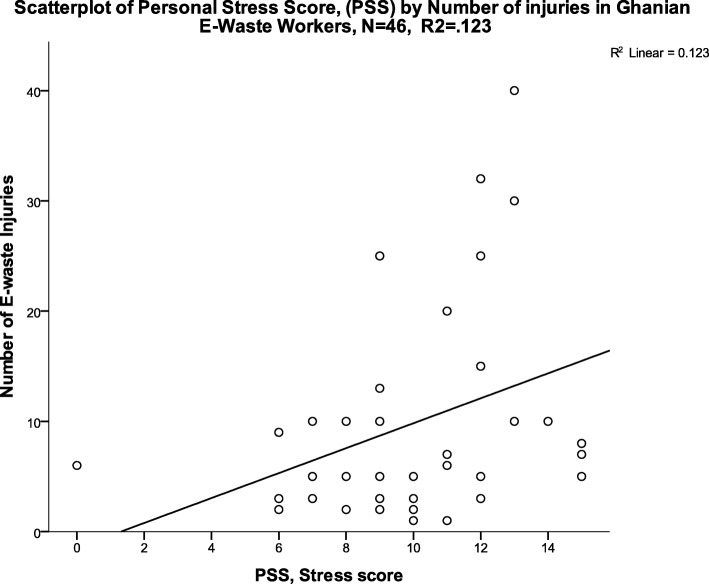
Perceived stress score vs. number of e-waste related injuries (R^2^ = 0.12, *p* = 0.01)

When all workers were considered, the Spearman correlation coefficient between PSS and injuries (Fig. 2) was significant, where higher stress levels were associated with more injuries (*r* = 0.42, *p* = 0.001). Age, self-reported health status, perceived noise exposure, and measured noise exposure were not significantly correlated with number of injuries. Age and stress were also not significantly correlated (data not shown).

### Poisson modeling

Our unadjusted Poisson regression results identified a number of variables that were significantly associated with number of injuries. These include: Age, education, perceived noise, impairment that limits work, measured noise, glove use, occupational stress score, perceived health status, perceived stress scale, and training prior to injury. Also, being interviewed in Dagbani and being interviewed by interviewer 2 were both significantly associated with an increased number of injuries. When all 46 participants were included in our adjusted multivariate Poisson regression model with number of injuries as the outcome (adjusted model, Table 4), higher PSS, not using gloves, and higher perceived noise exposure frequency were significantly associated with a higher number of injuries. Not performing dismantling work, greater age, and better self-reported health status were significantly associated with a reduced number of injuries. Interview language and interviewer were not selected in the forward stepwise selection process for the final adjusted model based on lack of improvement in the AIC when these variables were included in the model.

**Table 4:** Unadjusted (i.e., single predictor variable) and adjusted (i.e., multivariable) Poisson regression models with number of injuries as the outcome (*n* = 46 e-waste recycling workers)

Variable		Unadjusted			Adjusted (AIC = 397)		
	AIC	β	SE	*p*	β	SE	*p*
Intercept					2.30	0.34	< 0.001
Age (years)	476	−0.04	0.01	0.001	−0.03	0.01	0.008
Daily income^a^	489	−0.06	0.06	0.296	–	–	–
Duration of residence	488	−0.02	0.02	0.178	–	–	–
Education^a^	485	−0.12	0.06	0.036	–	–	–
Perceived noise^a^	480	−0.44	0.13	0.011	0.62	0.14	< 0.001
Impairment that limits work	464	0.67	0.17	< 0.001	–	–	–
Interviewer^a^	490	0.48	0.13	< 0.01	–	–	–
Interview language^a^	486	0.54	0.12	< 0.01	–	–	–
Married^a^	487	0.08	0.05	0.117	–	–	–
Noise TWA (dBA)	233	−0.05	0.01	< 0.001	–	–	–
Not dismantling^a^	487	−0.27	0.15	0.083	−0.41	0.16	0.009
Not using gloves^a^	462	0.59	0.13	< 0.001	0.46	0.13	0.001
Occupational Stress Scale (OSS)	434	0.06	0.01	< 0.001	–	–	–
Perceived health status	472	−0.18	0.04	< 0.001	−0.27	0.04	< 0.001
Perceived Stress Scale (PSS)	438	0.13	0.02	< 0.001	0.03	0.004	< 0.001
Received training prior to injury^a^	458	0.37	0.10	< 0.001	–	–	–

^a^Reference categories: Dismantling, wore gloves, low perceived noise, no formal education, interviewer 1, language other than Dagbani, unmarried, daily income < 10 GHS, no impairment that limits work, no training prior to injury

We completed a sensitivity analysis comparing the number of injuries associated with perceived noise exposure vs the measured noise levels from personal noise dosimetry (i.e., a multivariate Poisson model with the same variables in Table 4 but restricted to the subset of 26 participants with personal noise dosimetry). The subset of 26 workers did not differ significantly from those of the total sample for any of the variables assessed (data not shown). Our sensitivity analysis yielded model results which were generally very similar (data not shown); however, in this sensitivity analysis, higher measured noise levels were associated with a significant decrease in the number reported injuries (β = − 0.05 injuries per dBA, SE 0.01, *p* = 0.001).

## Discussion

This study is one of the first to evaluate injury risk factors among e-waste recycling workers. The participants in our study had experienced a substantial number of injuries in the 6 months prior to their participation in the study, many of them involving lacerations to the hand, a finding that has also been reported by other authors [9, 54]. Use of certain PPE, such as gloves, was found to be associated with a significantly reduced number of injuries. A number of factors, including participation in e-waste dismantling activities, higher perceived stress and more frequent perceived noise exposure, were associated with an increased number of injuries. These relationships remained strong even when controlling for other factors such as age, use of gloves, perceived health status, work activity, and measured noise levels. These findings suggest the need for increased attention to injury risks faced by e-waste recycling workers, and present possible opportunities for intervention, including increased public awareness, worker training programs, government intervention to address health and safety issues [46, 54], promotion of regulation, and government financing to enforce higher safety measures [52].

Although other studies have examined health-related issues among informal e-waste workers at the Agbogbloshie market site in Accra [23], our study filled an important gap by evaluating several upstream factors related to total worker health and safety outcomes. Our results supported our first hypothesis: greater perceived stress was associated with a higher number of injuries. Cohen’s PSS measures perceived lack of control over personal stress associated with day-to-day issues [21], and our results indicated that workers had high levels of perceived stress which could influence their injury rate related to e-waste recycling work. This finding, coupled with the significant independent relationship of self-reported health status and number of injuries, suggests that intervention efforts focused on mental and physical health among e-waste recycling workers may be warranted. Additional studies of the relationship between occupational exposures and working conditions among informal e-waste recycling workers and perceived and objectively assessed health status are needed to confirm these findings [2, 23] and better characterize the environmental health issues associated with e-waste recycling work [2, 7, 25].

Our second hypothesis (that increased noise exposure would be associated with a significantly increased number of injuries) was also supported by our results. Greater perceived noise exposure was associated with a significant increase in number of injuries after controlling for other co-exposures. However, in our sensitivity analysis, higher objectively measured noise levels (quantified via personal noise dosimetry) showed the opposite effect, where increase noised levels were associated with a significant reduction in the number of injuries. It is possible that this is a spurious finding, given the very small sample size of 26 participants for the sensitivity analysis. One important issue that may help explain these divergent results is that our evaluation of noise exposures was not temporally aligned with our injury evaluation, which may have introduced additional bias. Our noise measurements covered a single 24-h period (the maximum duration we deemed possible while still being able to reliably recover our dosimeters), while our perceived noise items addressed frequency of high noise exposure but did not specify a time period, and our injury questions related to injuries experienced in the past 6 months. Given this situation, the perceived noise exposure frequency variable may be considered a more valid measure of long-term noise exposure, since participant reporting may have involved consideration of exposures over a period of weeks or months. Our results, combined with those of others [16, 19, 33, 36, 37], support a relationship between occupational noise exposure and injuries, and suggest a potential route for interventions intended to reduce noise exposure and, perhaps, risk of injury.

The total number of injuries sustained by the e-waste workers in this study exceeded 400 over a 6-month period, including one worker who reported 40 injuries. Work activities, and particularly dismantling, were significantly associated with injuries even after controlling for other co-variates. Most of the injuries reported were lacerations from sharp objects and occurred during dismantling activities. While approximately one-quarter of the participating workers reported using gloves during e-waste recycling work, use of PPE was quite low overall. This likely reflects at least two factors: first, a lack of sufficient individual-level financial resources to individually purchase PPE (necessary, given the absence of a formal employer); and second, a likely lack of access to commercially-available supplies of PPE. However, we did not survey workers with regards to where they obtained gloves and other PPE, so there is substantial uncertainty regarding both PPE origin and pricing. Our results did, however, indicate that glove use was not significantly associated with age, income, or seniority. It is possible that the practice of handwashing before prayer (Wudo) in the Muslim tradition influences the decision to seek out protection for the hands. If worker’s hands are not defiled prior to prayer, they do not have to practice Wudo, which can be challenging given the lack of sanitation on the site. Therefore, it is possible that glove use may have more to do with religious practice than with occupational safety for at least some workers, in an effort to avoid having to purchase bottled or sachet water for Wudo.

In addition to injury hazards, participants reported long hours of work. Other studies have documented dangerous physical working conditions [3, 26, 39] and identified a relationship between mental health factors and occupational injury in other work settings [14, 28, 32]. Unfortunately, e-waste recycling workers at Agbogbloshie have previously reported insufficient income to afford the Ghanaian health insurance scheme, and as a result many of their injuries were left untreated [43, 54]. This is an important environmental justice issue; the informal e-waste recycling industry in Ghana has been reported to generate millions of dollars in revenue for the country annually [20], but the benefits to e-waste recycling workers have been limited. The economic opportunities e-waste recycling work presents for unskilled and often undocumented labor has likely hindered formalization of the work processes and, as a result, possibly slowed adoption of improved health and safety practices [29–31, 35, 40, 51, 53].

### Limitations

This study has a number of limitations. First is the small sample size and cross-sectional nature of the study. While most participants had worked at the site for several years, and anecdotal reports indicated that conditions and work practices do not appear to have changed substantially over that period, it is nevertheless possible that injury risks could have changed in meaningful ways over our 6-month injury reporting window. This could introduce positive or negative bias into our estimates of injury frequency. Also, because exposures and injury experiences were evaluated simultaneously, we were unable to assess causality; we cannot determine, for example, whether workers who reported poor health status have more injuries as a result of that health status, or vice versa. Second, our assessment of injury risk may have been influenced by the healthy worker effect, and our injury estimates may have been biased negatively by the exclusion of less healthy workers who are no longer doing e-waste recycling work due to illness or injury. Third, our assessment of work activities conducted by the workers was completed using activities reported to us by participants, that we then collapsed into smaller post hoc categories. We used this approach to try to reduce the large number of specific work activities reported by individual workers into a manageable number of broader job categories. However, in doing so, we may have introduced misclassification of work activities, which could bias our understanding of injury risks associated with our defined work activities. Fourth, our participants came from Northern Ghana and other West African countries and spoke a number of languages; it is possible that errors or miscommunication by our translators, differences in the context and interpretation of some questions, or social response biases introduced by perceived or actual social class differences between our participants and translators, may have introduced additional misclassification. This possibility is reflected in the significant univariate Poisson regression results for interview language and interviewer. The cultural appropriateness of the PSS and OSS instruments we used has not been validated in this population. Fifth, the temporal misalignment between our objective and subjective measures of noise exposure may at least partially explain the poor correlation between objective or subjective noise exposures, as well as the inconsistencies in our injury risk models that included a noise exposure variable. Finally, although we emphasized to participants that their responses would be confidential and would never be shared with others, some workers may have purposely misrepresented the number of injuries experienced or health status to avoid perceived adverse impacts on their employment.

## Conclusions

Our study is one of the first to evaluate injury experience among informal e-waste recycling workers and highlights the hazardous working conditions present at sites like Agbogbloshie. Our results suggest that these workers have an elevated risk of injury and experience reduced perceived health status and elevated levels of perceived and occupational stress. Additional studies with larger sample sizes and longitudinal study designs are needed to better characterize the injury risk among informal e-waste recycling workers. If future studies also demonstrate elevated risk of injury among these vulnerable workers, international programs and research efforts are needed to enhance the safety and economic sustainability of informal e-waste work.

## Abbreviations

AIC: Akaike Information Criterion
dBA: A-weighted decibels
LMIC: Low- or Middle-Income Country
OSS: Occupational Stress Scale
PPE: Personal Protective Equipment
PSS: Perceived Stress Scale
TWA: Time-Weighted Average
USD: United States Dollar
